# A simple consensus approach improves somatic mutation prediction accuracy

**DOI:** 10.1186/gm494

**Published:** 2013-09-30

**Authors:** David L Goode, Sally M Hunter, Maria A Doyle, Tao Ma, Simone M Rowley, David Choong, Georgina L Ryland, Ian G Campbell

**Affiliations:** 1 Peter MacCallum Cancer Centre, Sarcoma Genetics and Genomics Laboratory, St. Andrew’s Place, East Melbourne, Victoria, Australia; 2 Peter MacCallum Cancer Centre, Cancer Genetics Laboratory, St. Andrew’s Place, East Melbourne, Victoria, Australia; 3 Peter MacCallum Cancer Centre, Bioinformatics Core Facility, St. Andrew’s Place, East Melbourne, Victoria, Australia; 4 Centre for Cancer Research, Monash Institute of Medical Research, Monash University, Clayton, Victoria, Australia; 5 Sir Peter MacCallum Department of Oncology, University of Melbourne, Parkville, Victoria, Australia; 6 Bioinformatics Graduate Program, University of Melbourne, Parkville, Victoria, Australia; 7 Department of Pathology, University of Melbourne, Parkville, Victoria, Australia

## Abstract

Differentiating true somatic mutations from artifacts in massively parallel sequencing data is an immense challenge. To develop methods for optimal somatic mutation detection and to identify factors influencing somatic mutation prediction accuracy, we validated predictions from three somatic mutation detection algorithms, MuTect, JointSNVMix2 and SomaticSniper, by Sanger sequencing. Full consensus predictions had a validation rate of >98%, but some partial consensus predictions validated too. In cases of partial consensus, read depth and mapping quality data, along with additional prediction methods, aided in removing inaccurate predictions. Our consensus approach is fast, flexible and provides a high-confidence list of putative somatic mutations.

## Background

Massively parallel sequencing (MPS) of cancer exomes is becoming a commonplace technique, and has led to the identification of genes underlying the pathogenesis of a number of cancer types [1-6]. In response to the volume of data generated by these genome-scale studies, a host of software tools has been developed to aid in distinguishing genuine somatic mutations from germline variation, alignment artifacts, and inherent MPS errors [7-11]. The rarity and diversity of somatic events that occur on a background of tumor heterogeneity, normal contamination, technical artifacts, and genomic complexity makes this task particularly challenging [1,12].

Although the methodology applied by somatic mutation algorithms varies somewhat, the aim of each program is to identify tumor-specific variants by comparing sequence data from a tumor with that generated from a normal tissue (representing the germline) from the same patient (that is, matched normal DNA). The most common application is the identification of point mutations. The germline sample is usually assumed to be free of genetic material from the tumor, although this assumption can be tested and corrected for [12,13]. At every site where there are reads that differ from the reference genome, the probability that these reads contain legitimate genetic variants and not sequencing errors or technical artifacts is calculated. The probabilities for the tumor and germline data are compared, and a prediction about whether the site harbors a somatic mutation is made [7-11]. From this, a list of putative somatic mutations and associated confidence values is produced, which can be used in downstream analyses.

The choice of somatic mutation detection algorithm may have an important influence on the outcome of a tumor exome-sequencing study. Incorporating more information from a sequencing run (such as site-specific mapping and base qualities) improves the performance of variant detection over that of *ad hoc* metrics based on read counts alone [7,10,14,15]. Thus, it would be expected that predictions from different algorithms, which weigh different properties of the data in unique ways, may differ significantly. A conservative algorithm with high specificity may make very few incorrect predictions, but may miss many legitimate somatic mutations because of its low sensitivity. Similarly, a high-confidence set of somatic mutation predictions with a low false-positive (FP) rate is very useful in a clinical setting, but in a discovery-based research setting, it could limit the power to identify novel mutated genes and pathways [16]. This is important given the small number of recurrently mutated tumor driver genes, and the long list of infrequently mutated, yet biologically important, targets identified in many cancer types to date [17,18]. Conversely, although a more inclusive algorithm may have high sensitivity, the resolution of the follow-up analyses could be diminished by the inclusion of multiple FPs, which would also strain resources dedicated to validating mutations.

In most studies, only a single algorithm is used to predict which variants are somatic, with predicted mutations validated by an orthogonal sequencing technology, such as Sanger sequencing [2,4,6]. Although a number of published papers on somatic mutation detection programs have reported robust sensitivity and low error rates, the relative advantages of selecting one program over any other are not clear, especially as few programs have been evaluated extensively for their sensitivity and specificity in an unbiased fashion in independent tumor cohorts. A naive yet logical strategy could be to employ multiple somatic mutation prediction algorithms and to select variants for follow-up based on overlapping predictions, obviating the need to make *a priori* assumptions about the relative performance of each program. Such an approach assumes that true somatic mutations will show strong evidence of their existence in multiple programs, and that errors made by individual algorithms are unlikely to intersect.

We set out to address these questions by applying three publicly available somatic single nucleotide variant (SNV) detection programs, JointSNVMix2 [7], MuTect [8], and SomaticSniper [9]. All three implement sophisticated detection algorithms, and have been used in major tumor sequencing studies [2,8,19]. We used these three programs to make somatic mutation predictions for the exomes of 27 ovarian tumors and their matched germline samples. A subset of the predictions from each program was validated by Sanger sequencing. This high-confidence set of somatic point mutations and confirmed FP predictions was then used to assess the performance of each program and the relative benefit of combining predictions from multiple programs in a consensus approach, and to compare the properties of validated and miscalled somatic mutations.

## Methods

### Sample cohort

Ovarian tumors were collected through two primary sources: 23 cases from the Wessex region of southern England [20], and four cases through the Australian Ovarian Cancer Study (AOCS) [21]. The cohort consisted of 3 benign, 4 borderline and 10 invasive mucinous ovarian tumors, and 10 serous borderline ovarian tumors. Representative hematoxylin and eosin (H&E)-stained sections, and the tumor pathology reports were reviewed by pathologists for all cases included in the study.

### Ethics approval

The accrual of patient material used in this study was approved by the following human research ethics committees (HRECs): Southampton Hospital HREC, Peter MacCallum Cancer Centre HREC, and the HRECs of all centers participating in the AOCS. Informed consent was obtained from all patients included in the study. This project was reviewed and carried out in accordance with the ethical standards of the Peter MacCallum Cancer Centre HREC (Approval numbers 09/29 and 01/38).

### DNA extraction and preparation of exome-sequencing libraries

DNA extractions were performed as described previously [4,22]. Briefly, fresh-frozen ovarian tumors were needle-microdissected to enrich for epithelial cells, to a minimum of 70% epithelial cell content. DNA was extracted using the DNeasy Blood and Tissue Kit (Qiagen Inc., Valencia, CA, USA) according to the manufacturer’s recommendations. Matching peripheral blood DNA was collected and extracted from patients at the time of tumor collection, and used as the source of germline DNA.

To generate sequencing libraries, 500 ng to 1 ug of DNA were randomly sheared to approximately 200 bp using a Covaris S2 Ultrasonicator (Covaris, Woburn, MA, USA). Adapter ligated libraries were constructed and exome-enriched using either NEBNext DNA library preparation reagents (New England BioLabs, Ipswich, MA, USA) and the NimbleGen SeqCap EZ Human Exome Library (version 1; Roche Nimblegen, Madison, WI, USA) (non-indexed libraries) or TruSeq DNA Sample Preparation kits (Illumina, San Diego, CA, USA) and the NimbleGen SeqCap EZ Human Exome Library (version 2 Roche Nimblegen) (indexed libraries). Non-indexed libraries were each sequenced on one lane of an Illumina GAIIx (Illumina Inc., San Diego, CA, USA) sequencer, using 75 bp paired-end reads. Indexed libraries comprising three pooled samples were sequenced on one lane of a HiSeq 2000 sequencer (Illumina Inc.), using 100 bp paired-end reads. The mean coverage achieved ranged from 102 to 225 reads per site in the tumors and 119 to 188 reads per site in the germline.

### Exome sequencing read alignment and filtering

After quality based read trimming, sequence reads were aligned to the GRCh37/hg19 human reference genome using BWA [19] and duplicate reads marked using the Picard program [23]. Aligned reads for each tumor-germline pair were combined into one alignment file in binary sequence alignment (BAM) format [24], followed by local indel realignment and base quality recalibration using the Genome Analysis Tool Kit (GATK) software [14,25]. The GATK Unified Genotyper was used to identify putative SNVs within each individual tumor and germline sample.

### Identification of single nucleotide somatic mutations

MuTect (version 1.0.27783) was accessed through the MuTect website at the Broad Institute [8,26]. Because this was a development version of MuTect with limited options for setting parameters, only the default parameter set was applied, without filtering germline variants. Predictions not labeled as 'REJECT’ were accepted as confident somatic mutation predictions, and considered for subsequent downstream validation and analysis steps.

JointSNVMix2 (version 0.7.5) [7] was accessed through the JointSNVMix project site [27]. Model training and somatic mutation classification were performed using the 'joint_snv_mix_two’ algorithm. The default prior genotype probabilities provided in the JointSNVMix package were used for the training step. Predictions with a joint probability of having a variant in the tumor sample and no variant in the germline sample (p_AA_AB) of 0.9999 or greater were considered for subsequent downstream validation and analysis steps.

SomaticSniper (version 1.0.0) [9] was downloaded from the github project page for SomaticSniper [28] 0). Somatic SNVs were predicted using the joint genotyping mode (-J option) with the default prior probability of a somatic mutation (0.01). Reads with a mapping quality of 0 were filtered prior to somatic mutation identification. Predictions with a 'somatic score’ of 40 or greater were considered for subsequent downstream validation and analysis steps.

For each sample, the SAMtools mpileup tool [24] was used to extract data on the mapping qualities, directionality, and depth of reads that overlapped each SNV for which at least one program had made a somatic prediction. Putative somatic SNVs meeting the following criteria were considered for validation: 1) total read depth was of 8 or greater in both the tumor and matched germline; 2) mutant allele present at a frequency of 20% or greater in the tumor and 5% or greater in the matched germline; 3) mutant allele supported by read mapping in both the forward and reverse orientations (bidirectional); and 4) variant called in only one tumor (with the exception of the *BRAF* V600 and *KRAS* G12/G13 hotspot mutations, known to be frequently mutated in these tumor types), in order to eliminate alignment and germline coverage artifacts. These criteria were chosen to restrict validation to those variants within the sensitivity of Sanger sequencing, while also minimizing the inclusion of technical artifacts. For a small proportion of variants, unidirectional variants were also considered in order to provide some assessment of the value of considering only variants with bidirectional read support.

### Sanger sequencing validation

Prior to variant validation, 25 to 50 ng of DNA from each tumor sample were whole-genome amplified (WGA) using either the Repli-g Mini Kit or Repli-g Midi Kit (Qiagen). Putative somatic SNVs were validated by conventional Sanger-based sequencing analysis of PCR products obtained from the tumor WGA DNA. Purified PCR products were directly sequenced on an ABI3130 Genetic Analyzer using BigDye Terminator (version 3.1) sequencing chemistry (both Applied Biosystems, Foster City, CA, UDA). The somatic status of all validated mutations was confirmed by sequencing WGA DNA from the matching germline sample.

### Comparison of true positives and false positives

The R statistical software package (version 2.15) [29] was used to perform graphical and statistical comparisons of the data for predicted somatic mutations that were validated by Sanger sequencing (true positives; TPs) and predictions that did not validate or were germline variants (FPs).

To analyze the local sequence context around TP and FP predictions, a 100 bp region flanking each site was retrieved from the human genome (GRCh37 assembly) using version 64.37 of the Ensembl database [30]. Custom Perl scripts were used to assess the GC content, and the length and abundance of homopolymers flanking each site.

For each variant where validation was attempted, we counted the numbers of reads covering the variant site that were classed as uniquely mapped, multi-mapped, or mate-rescued by the XT field in the SAM file assigned by BWA. Mismatches were counted in these reads using the NM field in the SAM file.

## Results

### Somatic mutation prediction

We obtained somatic mutation predictions for each of the 27 ovarian tumor samples using MuTect, JointSNVMix2, and SomaticSniper. The thresholds for defining a putative mutation as somatic we attempted to set to be as equivalent as possible between programs, and thus chose a somatic score of greater than 40 for SomaticSniper, and a joint genotype probability (p_AA_AB | p_AA_BB) of greater than 0.9999 for JointSNVMix2. All MuTect predictions without a 'REJECT’ FILTER flag were considered in accordance with the developers’ guidelines. The input for all programs was a BAM file [24] with alignment data from BWA [31] and GATK IndelRealigner [14,25].

**Figure 1:**
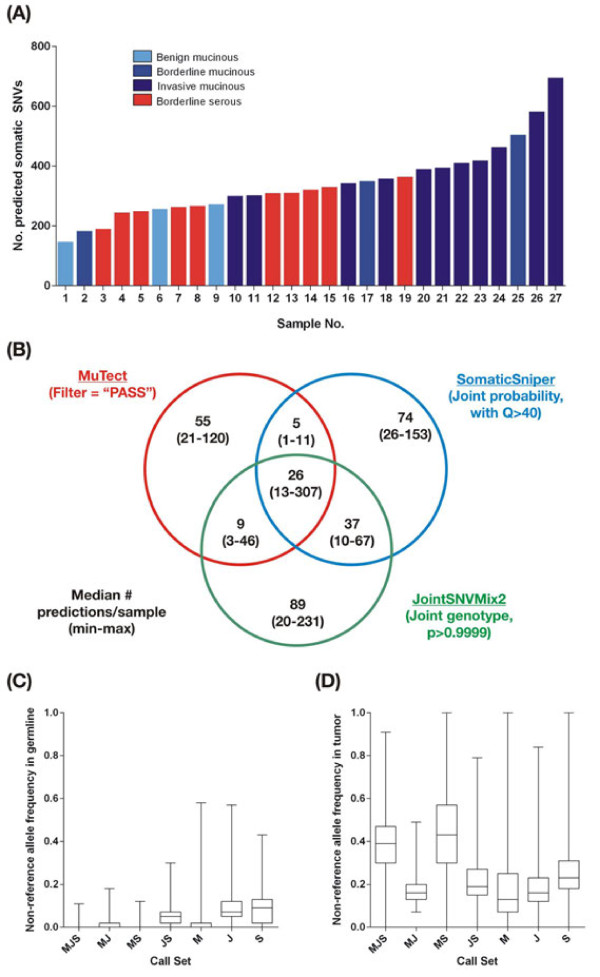
**Frequency, concordance and read depths of somatic variant predictions in 27 ovarian tumors. (A)** The total number of somatic SNVs predicted in each sample by the three algorithms. Bars are colored by tumor grade and histological subtype. **(B)** Concordance between somatic variant predictions from different algorithms. The minimum, maximum, and median numbers of predictions per 'call set’ per sample are shown. The filtering criterion used for each program is indicated. **(C)** Non-reference allele frequency, measured as the fraction of reads in the germline samples carrying the non-reference allele, at sites predicted to be somatic variants, within each call set. Each box covers the interquartile range, with a horizontal line representing the median. Whiskers indicate the minimum and maximum values. **(D)** Fraction of reads carrying the non-reference allele in the tumor samples at sites predicted to be somatic variants, within each call set. Call sets were defined by the programs that made a prediction above threshold using the following programs: M. MuTect, J = JointSNVMix2, S. SomaticSniper.

A combined total of 9,226 somatic SNV predictions were made by the three programs, with a median of 321 predictions per sample and a range of 147 to 695. There appeared to be an association between tumor grade and the number of predicted somatic mutations (Figure 1A), with invasive mucinous tumors harboring a higher number of predicted point mutations than benign or borderline tumors (Kruskal-Wallis. *P*<0.001). No association was found between estimates of tumor ploidy and normal contamination (as measured by ASCAT (Allele-Specific Copy number Analysis of Tumors [13])) or number of predicted somatic SNVs (see Additional file 1: Figure S1, Table S1, and Text S1). The proportion of non-reference reads (non-reference allele frequency; NRAF), was consistently and comparably low across samples (see Additional file 1: Figure S1C), indicating that the unfiltered mutation predictions contained significant numbers of FPs, which may be due to the somatic mutation detection algorithms included in this study not accounting adequately for tumor purity and ploidy (see Additional file 1: Text S2; see Additional file 1: Figure S12). Together, these finding suggest that the differences in the amount of somatic variation between the samples analyzed here is largely driven by biological factors. Benign tumors certainly could be anticipated to harbor fewer mutations, and similar inter-tumor variability has been reported from other studies [2,5,6], consistent with the notion that tumors at a more advanced stage of malignancy have acquired more mutations.

SomaticSniper and JointSNVMix2 generally predicted the most mutations per sample, with a median of 171 and 173 per sample, respectively. MuTect was more conservative, with a median of only 115 predictions per sample. However, the majority of somatic mutations predicted by any one algorithm were not confirmed by any of the other algorithms (Figure 1B). On average, the three programs agreed only on 11.5% of the somatic predictions for a given sample, while on average more than 80% of the somatic variants predicted in a single sample were identified by only one of the three programs used (see Additional file 1: Figure S2A). This trend remained even after non-coding variants were discarded (see Additional file 1: Figure S3). It is likely that a fraction of these program-specific predictions was accounted for by FPs. However, even if only a minority of such predictions is genuinely somatic, using only one program could potentially miss many genuine mutations.

### Comparison of the properties of mutation predictions from different algorithms

The poor consensus between the predictions made by each program may be explained in part by differences in their stringency settings; however, it is not clear how to directly normalize MuTect output to that of the other programs. Differences may also arise because of the distinct ways each program filters and processes sequencing reads between the tumor and germline samples, as all methods take into account base-calling and read-mapping accuracy measures when assigning probability to individual predictions.

To investigate further, we grouped the putative somatic mutations in all samples by the set of programs predicting a variant at that site (hereafter referred to as 'call sets’) and compared the number of reads containing the reference and non-reference allele (that is, an SNV or an 'alternate’ allele) at each site in each sample where a prediction was made (Figures 1C,D). The property that best discriminated between prediction sets was the frequency of reads with non-reference alleles in the germline samples. Predictions that were specific to SomaticSniper or JointSNVMix2 often fell at sites where a substantial number of reads with non-reference bases had mapped (Figure 1C). These results suggest that these two programs tolerate a larger number of non-reference alleles in the germline sample, which potentially introduces a significant number of germline variants into the call set, but such variants can be eliminated by downstream filtering on frequency of reads that do not match the reference. Conversely, MuTect is much more stringent on evidence for non-reference alleles in the germline.

Additionally, predictions made by one or two programs tended to have a lower proportion of non-reference reads (NRAF) in the tumor sample than predictions made by all three programs, for which NRAF was much higher (Figure 1D), with the exception of predictions that overlapped between MuTect and SomaticSniper. The low fraction of non-reference reads may have resulted in some sites not having sufficient allelic ratios to be predicted as putative somatic mutations by one or more algorithms, but enough support to rise above the thresholds of at least one program. Many of the differences between call sets are potentially the result of such 'borderline’, low-confidence predictions. For example, the Mutect-SomaticSniper call set has a higher median NRAF than the other two-algorithm call sets because JointSNVmix2 requires higher read depths to make confident somatic mutation calls (see Additional file 1: Figure S4) and SNV calls at low-depth sites tend to have higher NRAF because a minimum number of non-reference alleles are needed to make a prediction. Total read depths in the tumor and germline samples for each call set were not consistent with differences in NRAF between groups (see Additional file 1: Figures S4A-D), suggesting that NRAF is influenced by the tendencies of each program, and is not confounded by read depth thresholds.

### Validation of somatic mutation predictions

#### Enrichment for somatic variants

Although the variant callers used in this study were designed to identify somatic SNVs, a large number of germline variants and artifacts were anticipated to be present in each call set, based on previous analyses. We enriched for somatic SNVs using a set of minimal filters applied to all predicted SNVs. Sites covered by fewer than eight reads in the tumor and the germline samples were excluded, as were SNVs for which the non-reference allele frequency was greater than 0.05 in the germline sample or less than 0.2 in the tumor sample, and also variants that were identified in more than one sample (with the exception of the *KRAS* and *BRAF* oncogenic hotspot mutations). SNVs with evidence of the non-reference allele in the germline sample have a high probability of being FPs, such as germline variants or alignment or sequencing artifacts. However, considering only variants for which non-reference alleles are completely absent in the germline could result in some true somatic variants being discarded. Thus we selected an NRAF of 0.2 or higher in the tumor samples because our chosen validation method (Sanger sequencing) lacks sufficient resolution to robustly detect SNVs below this frequency [32].

Filtering had a disproportionate effect on the single-program call sets, removing 84 to 97% of the SNVs predicted by only one program, but removing less than 7% of SNVs predicted by all three programs (Table 1; see Additional file 1: Table S2). Filtering for the stated non-reference allele frequency in the tumor and germline had the greatest effect on variant numbers, indicating that the predictions unique to a single program are largely in regions with weak evidence for the presence of the non-reference allele in the tumor, and substantive evidence for the non-reference allele in the germline sample. As expected, this filtering increased the median percentage overlap in predictions for each sample (from 11.5% to 46.3%;, however, complete consensus predictions were still on average the minority of total predictions per sample (see Additional file 1: Figure S2B). We therefore anticipate the majority of the variants identified solely by a single caller to be low-confidence predictions, predominantly germline variants or sequencing and alignment artifacts.

**Table 1:** **Total variant calls made by each caller, pre-filtering and post-filtering out variants not suitable for validation by Sanger sequencing**^**a**^

	**MJS**	**MJ**	**MS**	**JS**	**M**	**J**	**S**	**Total**
All SNVs predicted, n (% of all variants)^b^	1,483 (16.1%)	83 (0.9%)	462 (5.0%)	298 (3.2%)	1,756 (19.0%)	2,387 (25.9%)	2,757 (29.9%)	9,226
SNVs suitable for validation (% of all SNVs suitable for validation)^c^	1385 (54.4%)	16 (0.6%)	370 (14.5%)	57 (2.2%)	279 (11.0%)	80 (3.1%)	360 (14.1%)	2,547
Average number (range) of filtered SNVs per sample	51.3	0.6	13.7	2.1	10.3	3.0	13.3	94.3
	(1 to 246)	(0 to 6)	(5 to 42)	(0 to 11)	(3 to 33)	(0 to 23)	(5 to 25)	(38 to 321)
Number (%) of SNVs that could not be used for validation	98 (6.6%)	67 (80.7%)	92 (19.9%)	241 (80.9%)	1477 (84.1%)	2307 (96.6%)	2397 (86.9%)	6679 (86.9%)

*Abbreviations:* J. JointSNVMix2; JS, JointSNVMix2 + SomaticSniper; M, MuTect, MJ, MuTect + JointSNVMix2; MJS, MuTect + JointSNVMix2; + SomaticSniper; MS, MuTect + SomaticSniper; NRAF, non-reference allele frequency; S, SomaticSniper; SNV, single nucleotide variant.

^a^Call sets are identified as described in Figure 1.

^b^Predicted variants were filtered on the basis of read depth and NRAF, as described in the text.

^c^'Suitable for validation’ means evidence for SNV met criteria for validation by Sanger sequencing (tumor NRAF ≥0.2, germline NRAF ≤0.05 and ≥8 reads in both samples.

#### Variant selection and validation

To determine what fraction of predictions in each call set were FPs, the predicted somatic SNVs were divided into call sets according to which program(s) identified them. Subsets of putative SNVs were selected from each group for validation by Sanger sequencing. The variants selected were biased towards those in coding regions, as these are currently of the greatest clinical and scientific interest. Some non-coding variants were also investigated to obtain robust numbers in each call set; these variants demonstrated very similar call set trends and characteristics to coding variants (see Additional file 1: Figures S3 and S4).

A total of 364 predicted somatic SNVs were selected (Table 2). Our aim was to assess the accuracy of somatic mutation predictions made by each program, and of the predictions in each call set as well, to determine if consensus predictions based on multiple algorithms are more reliable than those made by a single program. We calculated the positive predictive value (TP/(TP+FP)) to assess specificity, and the true positive rate (TP/)TP+FN)) to estimate sensitivity.

**Table 2:** Validation results by call set

	**MJS**	**MJ**	**MS**	**JS**	**M**	**J**	**S**	**Overall**
Variants assessed^a^	181	13	37	28	31	26	48	364
**True positives**^b^**(% true positive)**	**179 (98.9%)**	**5 (38.5%)**	**29 (78.4%)**	**10 (35.7%)**	**4 (12.9%)**	**1 (3.8%)**	**1 (2.1%)**	**229 (62.9%)**
Germline^c^	1	0	5	3	15	4	11	39
Did not validate^c^	1	8	3	15	12	21	36	97

*Abbreviations:* J, JointSNVMix2; JS, JointSNVMix2 + SomaticSniper; M, MuTect, MJ, MuTect + JointSNVMix2; MJS, MuTect + JointSNVMix2; + SomaticSniper; MS, MuTect + SomaticSniper; NRAF, non-reference allele frequency; S, SomaticSniper; SNV, single nucleotide variant.

^a^Number of sites that were successfully amplified and Sanger sequenced.

^b^Indicates the number of SNVs that were confirmed as somatic.

^c^False-positive SNVs were either detected in the matched germline (normal) sample ('Germline’) or did not validate in the tumor sample ('Did not validate’).

#### Validation rates

Of the 364 predicted SNVs assessed by Sanger sequencing, 229 represented genuine somatic variants. Somatic mutations predicted by all three programs (n = 181) had the highest validation rate (98.9%) (Table 2), showing that the full consensus between three somatic mutation detection programs provides a very high-confidence list of predicted somatic mutations. This high validation rate was achieved with minimal read depth and allele frequency filtering, and no additional filtering using often-employed properties, such as bidirectionality of reads and variant quality scores. The vast majority (97%) of the variants covered only by unidirectional reads fell outside the full consensus call set (see Additional file 1: Table S3), however, all four variants assessed from the full consensus call set with unidirectional read support were confirmed as genuine somatic mutations. In the full consensus call set, variants with GATK quality scores as low as 58 [14,25] were also validated as somatic.

Overall, 56.4% of the somatic mutations identified by two of the programs were also validated (Table 2), although the validation rate varied from 35 to 78% according to which two programs were considered. Predictions that overlapped between MuTect and one other program were more accurate than those found by the intersection of JointSNVMix2 and SomaticSniper predictions. Conspicuously, variants in the overlap of MuTect and SomaticSniper, where 78.4% of variants represented genuine somatic SNVs, had notably lower average read depths compared with variants called by JointSNVMix2 (see Additional file 1: Figure S4). This more stringent requirement for read depth probably contributes to high-quality, true somatic variants with lower read coverage being discarded by JointSNVMix2, shifting them from the full consensus call set to the MuTect-SomaticSniper partial-consensus call set (see Additional file 1: Table S5). Predictions made by two programs had a much higher validation rate than those made by a single program alone; only 6 of 105 (5.7%) SNVs predicted by one program could be validated (Table 2), indicating that predictions that are not supported by multiple programs are largely artifactual. The level of normal contamination was observed to affect both FP and TP rates, both of which improved as tumor purity increased, largely due to improved ascertainment of TPs as a result of reduced normal contamination (see Additional file 1: Figure S1D).

MuTect and SomaticSniper had comparable sensitivity in our dataset, as they both found approximately the same proportion of the 229 TP mutations that were ultimately confirmed, while the sensitivity of JointSNVMix2 was 10% lower (Table 3). MuTect showed the highest specificity (82.8%), as it had 4% and 8% higher specificity than SomaticSniper and JointSNVMix2, respectively, for the putative somatic SNVs selected for validation (Table 3), when predictions were combined across call sets. However, the performance of a given program will depend on the arbitrary threshold chosen for accepting a prediction.

**Table 3:** Summary of validation results by program and combination of programs

**Call set**	**Specificity**	**Sensitivity**
MuTect	82.8%^a^	94.8%^b^
JointSNVMix2	78.6%^a^	85.2%^b^
SomaticSniper	74.5%^a^	95.6%^b^
Consensus of 3 programs	98.9%^c^	78.2%^d^
**Consensus of 2+ programs**	**86.1%**^c^	**97.4%**^d^
All variants	38.6%^c^	100%^d^

*Abbreviations:* TP, true positive.

^a^Number of TPs predicted by program/all predictions by program for which validation was attempted.

^b^Number of TPs predicted by program/all TPs.

^c^Number of TPs predicted in the group/all predictions by program for which validation was attempted.

^d^Number of TPs predicted in the group/all TPs.

Although variants predicted by all three algorithms had the highest specificity (Table 3), 46% of the filtered variants fell outside of the three-program consensus, and the overall validation rate for variants detected by fewer than three programs was greater than 25%. Thus, considering only consensus predictions will miss a considerable number of genuine somatic SNVs. This demonstrates how improved accuracy in somatic mutation prediction can have costs in terms of limited detection sensitivity, suggesting that thresholds need to be tuned to strike a balance between specificity and sensitivity. For example, the specificity of predictions made by at least two or more programs was superior to that of any one program alone, without the same reduction in sensitivity seen in the full consensus set (Table 3).

### Distinguishing false positives from true positives

TP variants identified by one program alone may not be readily distinguishable from FPs, and therefore it is of questionable value to pursue such variants. Given that follow-up validation of predicted variants by Sanger sequencing is labor-intensive and expensive [32], a robust set of criteria for prioritizing putative point mutations for validation, particularly those lacking consensus between prediction algorithms, would be of great benefit. To this end, we analyzed the validation cohort in more detail to identify factors distinguishing TPs from FPs. We focused on properties that measure variant representation, base quality, and sequence and mapping contexts. These metrics were reported by SAMtools mpileup and BWA, and thus were measured in a consistent fashion for all predictions, independently of what was reported by the three programs used. Although over a dozen unique properties were examined (see Additional file 1: Text S3; see Additional file 1: Table S4) we focused on two that were found to correlate strongly with validation status: sequencing coverage and read-mapping statistics.

#### Sequence coverage and allele frequencies

**Figure 2:**
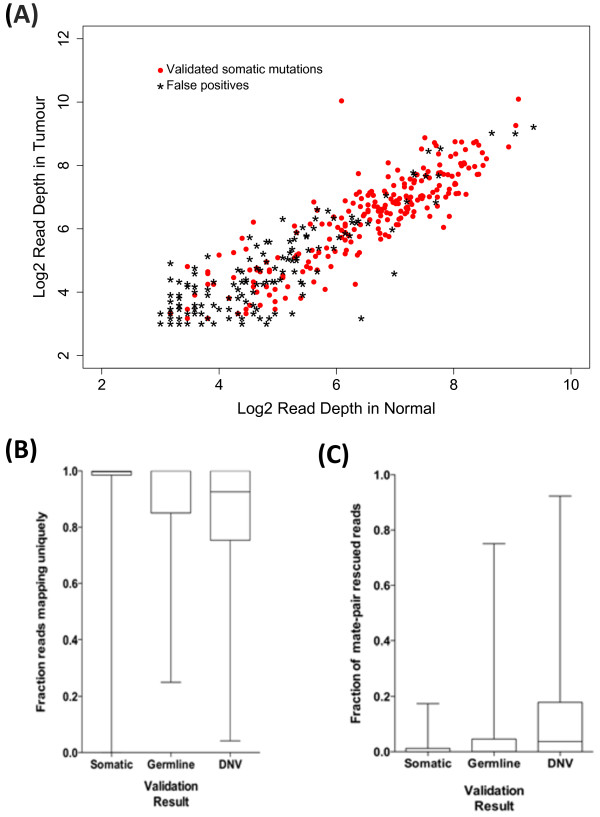
**Sequencing coverage and read-mapping results for validated somatic mutations and false positives. (A)** Log2-scaled read depths for sites harboring validated somatic mutations (red dots) and mutations that failed validation (black stars), in the tumor and normal samples from each individual predicted to harbor the mutation. **(B)** Fraction of reads containing the non-reference allele that mapped uniquely by BWA for validated somatic mutations (Somatic), mutations that were also detected in the germline sample during validation (Germline) and those that were not detected in either the tumor or the germline during validation (Did Not Validate; DNV). **(C)** Fraction of reads mapped by BWA using mate-pair rescue for validated somatic mutations (Somatic), mutations that were also detected in the germline sample during validation (Germline) and those that were not detected in either the tumor or the germline during validation (DNV). **(B**,**C)** Each box covers the interquartile range, with a horizontal line representing the median. Whiskers indicate the minimum and maximum values.

Sequence coverage was a major indicator of the accuracy of a predicted mutation. FP somatic mutation predictions had significantly lower total read depth, in both the tumor and the germline sample (Table 4; Figure 2A; see Additional file 1: Figures S6 and S7A). This discrepancy was seen in both alleles, and was conserved within the set of non-consensus predictions (see Additional file 1: Figure S7A). Another factor that correlated strongly with validation status was the non-reference allele frequency in the tumor sample, which showed differences between FPs and TPs (Table 4; see Additional file 1: Figure S6B and S7B), pointing to the relative proportion of alleles as another key metric in estimating the confidence of a somatic mutation prediction.

**Table 4:** Sequencing coverage and read-mapping quality features for true-positive and false-positive predictions

**Feature**	**All TPs**^**a**^	**All FPs**^**b**^	**DNV**^**c**^	**Germline**^**d**^
Median read depth				
Germline	105	25^h^	28^h^	12^g^
Tumor	94	20^h^	21^g^	19^g^
Mean non-reference allele frequency				
Germline	0.08%	1.4%^g^	1.27%^g^	1.77%^g^
Tumor	43.10%	30.9%^h^	27.3%^h^	39.80%
Mean percentage uniquely mapped reads	97.10%	85.3%^f^	83.7%^f^	89.30%
Fraction of SNVs with <95% reads mapping uniquely	8.3% (19/229)	52.2%^h^ (71/136)	57.7%^h^ (56/97)	38.4%^f^ (15/39)
Fraction of predicted SNVs with >5% of reads mate-rescued	4.4% (10/229)	39.7%^h^ (54/136)	46.3%^h^ (45/97)	23.1%^e^ (9/39)
Mean percentage of reads mapped to multiple locations	1.90%	4.7%^e^	4.40%	5.5%^f^

*Abbreviations:* DNV, Did not validate; FP, false positive; J, JointSNVMix2; JS, JointSNVMix2 + SomaticSniper; M, MuTect, MJ, MuTect + JointSNVMix2; MJS, MuTect + JointSNVMix2; + SomaticSniper; MS, MuTect + SomaticSniper; NRAF, non-reference allele frequency; S, SomaticSniper; SNV, single nucleotide variant.; TP, true positive.

^a^'Includes all SNVs that validated in the tumor sample.

^b^Combined value for these two categories of FPs.

^c^ Indicates SNVs that were not confirmed in the tumor sample.

^d^Indicates SNVs that were also detected in the matched germline.

Significance was tested using Wilcoxon rank-sum test for continuous variables and Fisher’s exact test for fraction of reads mapping uniquely/by mate-pair rescue/to multiple locations: :^e^*P*<0.05; ^f^*P*<1 × 10^-5^; ^g^*P*<1 × 10^-10^;,,^h^*P*<1 × 10^-15^.

#### Read mapping

Errors produced by inaccurate mapping and the misalignment of short sequencing reads can create the appearance of apparent sequence variants that do not actually exist [14,33]. To investigate the extent to which this effect contributed to FPs in our validation set, data on the number of unique mapping reads and on the number of reads mapping to repeat regions were extracted from the mpileup files for each sample. On average, FP mutations had a lower fraction of uniquely mapping reads than did genuine somatic SNVs (Table 4, Figure 2B). Thus, inaccurate or incorrect read mapping is a likely source of the FPs in our dataset. This association was stronger for somatic SNVs that did not validate (DNV) in the tumor sample, where 61.8% of SNVs had less than 99% of their reads map uniquely, compared with 73/229 (31.8%) of the validated SNVs (Fisher exact test; *P*<6.8 × 10^-7^), than it was for SNVs that were also found in the germline sample ('Germline’) where only 18/39 SNVs had <99% uniquely mapped reads (46.2%; Fisher exact test *P* = 0.09).

One method used by the BWA alignment process to establish the correct location of an ambiguously mapping read is to attempt alignment within a limited region defined by the mapping location of the other read in the mate pair [31]. Such 'mate-rescued’ reads made up a significantly higher proportion of the reads for the SNVs that did not validate, compared with the genuine somatic SNVs (Table 4, Figure 2C). This trend was stronger in the DNV predictions, as 46.4% of SNVs in that category had more than 5% of their reads mapped using mate-pair rescue, compared with 4.4% of validated SNVs (Fisher exact test. *P*<2.2 × 10^-16^), while 23.1% of predicted SNVs in the 'Germline’ category had greater than 5% mate-rescued reads (Fisher exact test, *P* = 0.0038). It appears the process of mate- escue can falsely generate sites that differ from the reference sequence, perhaps by forcing misalignment of reads as a result of constraints introduced by the mapped location of their mate pair.

Reads from common sequence repeats are often difficult to map uniquely, given the numerous potential high-scoring matches such reads can have. FP SNVs were more likely than validated variants to have reads that mapped to multiple locations, but in contrast to uniquely mapping reads, this effect was strongest for SNVs in the 'Germline’ category (Table 4; see Additional file 1: Figure S8), pointing to possible differences in the underlying causes of these two different classes of FPs. Misalignment of non-unique reads could lead to apparent FPs in the tumor sample, while SNVs may be missed in the germline samples if reads are being titrated away by mismapping to similar sequences elsewhere in the genome. The latter effect could be particularly pronounced when a read maps equally well to two locations in the genome, as BWA and other read mappers randomly map such reads to one of the two locations [31].

### Additional filtering of partial-consensus predictions

The accuracy of predictions lacking full consensus between programs is often low (2 to 36%, excluding the MuTect-SomaticSniper call set), but ignoring these SNVs could result in potentially important genuine somatic mutations being discarded. Although many factors that differ between TPs and FPs can be found, identifying filters that can be readily implemented by researchers to significantly enrich for TPs without sacrificing sensitivity is complex. Because FP calls arise for a variety of technical and biological reasons, as well as stochastically, no single feature or simple agglomeration of features is able to separate 100% of TPs from FPs. However, the validation rates from the two-caller overlaps are sufficient to warrant investigation as to whether further filtering and selection can enrich for TPs within these groups.

We tested the effects on the initial (base) validation rate and loss of TP variants in applying additional filtering parameters based on thresholds for sequencing coverage, non-reference allele frequency, and mate-rescued reads. These variants were selected based on their strong association with validation status (Table 4), and threshold values that best optimized discrimination of TPs from FP predictions were applied to the partial-consensus and no-consensus call sets.

The single filter that best enriched for TPs was percentage of reads mapped using mate-pair rescue. Simply removing SNVs for which 7% or more of the mapped reads were mate-rescued increased the validation rate in the lower confidence prediction groups from 27% to 38% without eliminating any TPs (Table 5). Decreasing the germline non-reference allele frequency from less than or equal to 0.05 to less than or equal to 0.02 also increased the validation rate with a low effect on TPs (Table 5). Other metrics were not as effective. Increasing the minimum read depth in the tumor and germline from 8 to 10 reads resulted in a 5% increase in the validation rate, but at a cost of 10% of TPs. Further increases in read depth thresholds rapidly escalated the TP dropout rate (see Additional file 1: Table S5). Increasing the minimum tumor non-reference allele frequency was explored, but was also found to excessively penalize TPs (see Additional file 1: Table S5). Filtering variants based on whether they were represented by bidirectional reads was also explored, but found to have little effect on the validation rate (see Additional file 1: Table S5).

**Table 5:** Additional filtering improved specificity of predictions lacking full consensus

	**Validation rate**^**a**^			**TP dropout rate**^**d**^
	**Partial consensus**^**b**^	**No consensus**^**c**^	**Overall**	
Base validation rate^e^	44/78 (56%)	6/105 (6%)	50/183 (27%)	
Percentage of mate-rescued reads <7%^f^	44/64 (64%)	6/69 (9%)	50/133 (38%)	0%
RD >10 (tumor and germline)^g^	40/68 (59%)	5/72 (7%)	45/140 (32%)	10%
Germline non-reference allele frequency ≤0.02^h^	42/65 (65%)	6/75 (8%)	48/140 (34%)	4%
GATK prediction for SNV in tumor^i^	43/66 (66%)	5/47 (11%)	48/113 (42%)	4%
GATK + mate-rescued	43/56 (77%)	5/31 (16%)	48/87 (55%)	4%
GATK + mate-rescued + RD >10	39/46 (85%)	4/19 (21%)	43/65 (66%)	14%

*Abbreviations:* RD, read depth; SNV, single nucleotide variant; TP, true positive.

^a^Validation rate = TPs/total SNVs assessed.

^b^Partial-consensus predictions (made by two programs).

^c^No consensus predictions (made by only one program).

^d^Percentage of TPs that would be discarded if the indicated set of filters was applied, that is, loss in sensitivity.

^e^Refers to specificity prior to filtering.

^f^Filtering on percentage of reads made from mate-pair rescue.

^g^Filtering on RD increased from 8 to 10 in tumor and germline.

^h^Non-reference allele frequencies in germline decreased from ≤0.05 to ≤0.02.

^i^Filtering SNVs predicted in the tumor but not the germline by GATK Unified Genotyper.

We also examined the value of increasing the overlap between the original three programs by lowering the stringency of the parameters used for the initial call sets, thereby including some variants that were initially discarded by the third program. A large proportion of the SNVs identified by two programs at or above our initial thresholds was in fact also detected by the third program, but below threshold. Although this approach significantly increased the validation rate from 27% to 63%, the effect on TP dropout was also significant (34%) (see Additional file 1: Table S6).

Based on our findings, a simple approach for improving the reliability of lower confidence somatic mutations would be to implement an additional program. GATK Unified Genotyper [14,25] is optimized for variant calling on individual or pooled samples; however, it can be (and regularly is) used to find somatic mutations, by comparing the set of confident variant predictions between a tumor sample and its matched germline control. Taking the consensus between GATK predictions and one or two of MuTect, JointSNVMix2, and SomaticSniper increased the overall specificity in these lower confidence groups from 27% to 42%, while discarding only two TPs (Table 5). In combination, consensus with GATK Unified Genotyper and mate-pair rescue read filtering increased specificity to 55%. Additionally, requiring a minimum read depth of 10 in both the tumor and germline further increased specificity to 66%, but this gain must be weighed against the 14% loss in sensitivity that resulted from applying these filters (Table 5). The results of additional filtering on each call set are provided (see Additional file 1: Table S5).

## Discussion

We have presented here an easily implemented but highly effective strategy for enriching true somatic SNVs in exome-sequencing data, using consensus between multiple somatic mutation prediction algorithms. Our validation data demonstrates how a consensus approach greatly improves specificity while preserving high sensitivity. Such an approach can provide guidelines for the interpretation and prioritization of predicted somatic variation from matched tumor-germline exome-sequencing data for downstream validation studies (Table 6).

**Table 6:** Recommendations for applying consensus results to somatic mutation predictions

**Prediction set**	**Recommendations**
Consensus	Accurate, high-confidence predictions suitable for further analysis
Partial consensus	Roughly equal numbers of genuine somatic mutations and false positives. Utility dependent on need to maximize sensitivity. Further filtering can be performed to improve confidence
No consensus	Largely false positives; may be disregarded unless compelling biological interest exists. Explore using high-confidence list of variants from consensus

The way this consensus approach should be applied will ultimately rest on the particular needs and aims of a given project. For example, in a clinical setting, where the priority is for maximum accuracy, simply taking the complete consensus set of variants might be sufficient. By contrast, the benefits of improved sensitivity may outweigh the burden of extra validation in certain contexts. In addition, a longer list of mutations increases power when searching for the enrichment of mutations in genes or pathways [16], and thus in such situations it may be better to consider variants with incomplete consensus.

Clearly, complete consensus between three somatic mutation prediction programs provides a very high-confidence set of predicted mutations. Conversely, predictions made by a single program, and not replicated by another, could be considered FPs, unless there is a compelling biological reason to pursue validation. For variants with partial consensus, further value can be derived from these calls by considering additional characteristics. The three programs used here were selected because they are widely used in the cancer MPS community, and use distinct detection algorithms, but any combination of programs should lead to improved somatic mutation detection. Consistent with our findings, requiring consensus with additional prediction algorithms improved specificity, while minimizing the loss of TPs.

Introducing additional filtering based on read-mapping statistics further improved specificity without reducing sensitivity, in particular with filters based on the fraction of reads that were mate-pair rescued. This is, to our knowledge, the first report linking the often-used process of read mapping by mate-pair rescue as a source of FP variant calls in MPS. Although filtering on read depth can provide minor improvements to the TP rate, the cost in sensitivity may be substantial. Likewise, the effects of sample purity and ploidy need to be considered when filtering predictions having low frequencies of non-reference reads. TP rates could be raised further by increasing the stringency of filtering on evidence for the non-reference allele in the germline, and by filtering germline SNVs using public resources such as the Exome Variant Server [34] and the 1000 Genomes database [35]. Such filtering was not performed here, as the aim was to assess the raw output from the somatic variant prediction algorithms.

Our findings show how applying just a few simple heuristic filters dramatically improves discrimination between tPs and FPs for partial-consensus predictions, suggesting a means by which somatic point mutation prediction algorithms could be further improved. More sophisticated somatic mutation filtering methods do exist, and could be used to improve classification further. For example, Ding *et al.* showed that applying machine learning algorithms based on predictive discriminative approaches to a set of 106 features based on sequencing metrics significantly improved on existing methods of somatic mutation detection [36]. Similarly, Lower *et al.* used a scheme based on sequencing of control samples in duplicate, and applied a random-forest classifier based on the output of three somatic mutation prediction programs (including one of those used here, SomaticSniper) to develop a false-discovery rate (FDR) measurement that they showed can be used to enrich for true somatic variants [37]. However, these approaches rely on generating extra, redundant sequencing and/or complex implementation of advanced algorithms, which may be beyond the means of some laboratories, and might hamper retrospective study of existing data. By contrast, obtaining a consensus set of predictions can be quickly and easily implemented using currently available software. This approach avoids the need for complex, highly specialized software or the generation of additional datasets, and provides specificity and sensitivity suitable for most purposes. Indeed, most of the 'low-FDR’ mutations validated by Lower *et al.* were consensus predictions between the three programs they used on their data [37]. Thus, we feel our approach should be accessible to a wide array of cancer researchers, even those without access to advanced bioinformatics resources.

The success of a consensus approach is also expected to vary with the sensitivity and accuracy of the somatic mutation prediction algorithms. We selected three programs that are widely used and considered 'state of the art’ for their accuracy and sensitivity, but any set of available programs for somatic mutation prediction could be used, and would be likely to share some fraction of their predictions [10,37]. Very similar algorithms would be expected to have a high degree of overlap in their predictions, while less similar algorithms would in general be expected to show poor overlap; however, some level of dissimilarity is necessary for the consensus approach. Although it may be anticipated that sequencing artifacts (arising from features such as sequence context, GC content, capture bias, and alignment artifacts) would affect the tumor and germline sample equally, and thereby result in consensus FPs, this is evidently not always the case. Differences in the quality of the tumor and germline DNA, allelic imbalances in the tumor, and stochastic allelic dropout in regions of low read depth are likely to unveil artificial 'somatic’ variants, to which each variant calling program is more or less sensitive. Users should adjust program parameters and downstream filtering accordingly, as optimal thresholds will vary by dataset and experiment. One strength of a consensus approach is that can be applied to assign confidence to predictions, even when there is uncertainty over the best cut-off thresholds to use.

Our study is limited by the validation approach taken, in that the validation rate may be affected by a combination of the sensitivity of Sanger sequencing and the inaccuracy in the allele frequencies generated by MPS, as the frequencies are an approximation, which is influenced by multiple PCR steps and capture efficiency. However, all of the samples and call sets used will have been influenced by these factors. Additionally, as this was a discovery dataset and the full set of true somatic mutations in our samples is unknown, the true sensitivity and specificity rates cannot be determined and the reported TP rate is a function of the somatic mutation detection programs used. Furthermore, we could not assess the usefulness of our consensus approach for rare somatic variations, as detection by Sanger sequencing is limited to variants with allele frequencies of greater than 20% [32].

Our results are most directly relevant to exome capture sequence data. The expected number, overlap, and reliability of somatic predictions may vary in situations with much higher average coverage (such as targeted resequencing of selected regions) or lower average coverage (such as whole-genome sequencing). However, we anticipate that even in the aforementioned situations, confidence would still be increased by the inclusion of multiple prediction algorithms.

Although we did not explore the validation rates of variants with allele frequencies less than 0.2, because of the technical limitations of the chosen validation method [32], these variants are of interest to many researchers [11]. It is possible that a simple consensus process may also work well to enrich for true somatic variants in this more difficult area. The characteristics and inbuilt thresholds of the individual program(s) used for variant calling can differ significantly, and need to be matched to the variants of interest.

## Conclusions

Taking the consensus of somatic SNV detection programs was found to be a powerful method for increasing the validation rate while maintaining maximum sensitivity. Although each program aims to identify true somatic variants and thus they have overlapping strategies, each has also been designed using independent methods resulting in program-specific limitations. The consensus approach compensates for this to some degree, allowing each program to reaffirm the predictions made by the others, and improving confidence by removing many of the false predictions generated by data artifacts. Similar effects are likely to influence other bioinformatics classification problems, and thus, while this approach is somewhat simplistic, it may prove effective for a variety of genomics and bioinformatics analyses.

## Abbreviations

ASCAT: Allele-specific copy number analysis of tumors; GATK: Genome analysis tool kit; J: JointSNVMix2; JS: JointSNVMix2 + SomaticSniper; M: MuTect, MJ, MuTect + JointSNVMix2; MJS: MuTect + JointSNVMix2 + SomaticSniper; MPS: Massively parallel sequencing; MS: MuTect + SomaticSniper; NRAF: Non-reference allele frequency; S: SomaticSniper; SNV: Single nucleotide variant.

## Competing interests

The authors have no competing interests to declare.

## Authors’ contributions

DG, SH, MD, GR, and IC designed the study and wrote the manuscript. DG and MD generated the somatic mutation predictions for all samples. SH, GR, SR, and DC prepared samples for exome sequencing and carried out validation of predicted variants. DG, SH, MD, TM, and GR analyzed the somatic mutation prediction and validation data. All authors read and approved the final manuscript.

## Supplementary Material

Additional file 1: Text S1 ASCAT (Allele-Specific Copy number Analysis of Tumors) analysis of tumor ploidy and purity. **Text S2**. Variant allele frequencies and tumor purity. **Text S3**. Distinguishing true somatic mutations from false positives using base quality, strand bias and local sequence context. **Table S1**: The exome cohort used in this study. **Table S2**: Amount of coding and non-coding variation in each call set, before and after filtering. **Table S3**: Fraction of sites with unidirectional reads per call set. **Table S4**: Base quality and sequence context for true and false positives. **Table S5**: Filtering Single nucleotide variants.(SNVs) using Genome Analysis Tool Kit (GATK) results, percentage mate-pair rescued reads and read depth. **Table S6**: Partial consensus predictions when the predictions from the third program was below threshold. **Figure S1**: Number of somatic mutation predictions as a function of tumor ploidy and aberrant cell fraction. **Figure S2**: Percentage overlap in somatic mutation predictions per sample. **Figure S3**: Percentage of SNV predictions in each call set for all variants, coding and non-coding. **Figure S4**: Read depth and allele frequency characteristics for coding and non-coding SNVs in each call set. **Figure S5**: Read depth and non-reference allele frequency for true and false positives, for all assessed variants. **Figure S6**: Read depth and non-reference allele frequency for true-positive and false-positive partial-consensus and unique predictions. **Figure S7**: Fraction of reads mapping to repetitive sequences for true somatic mutations and false positive predictions. **Figure S8**: Base qualities for true somatic mutations and false positives. **Figure S9**: Strand bias for true somatic mutations and false positives. **Figure S10**: GC content for true somatic mutations and false positive predictions. **Figure S11**: Homopolymer content for true somatic mutations and false positives. **Figure S12**: Influence of false-positive rates, estimated ploidy, and estimated aberrant cell fraction on number of predicted somatic mutations. Click here for file
