# Supplementary material for: A simple consensus approach improves somatic mutation prediction accuracy
Source: Genome Med. 2013 Sep 30;5(9):90. doi: 10.1186/gm494 (PMC3978449; doi:10.1186/gm494)
Supplement: Additional file 1: Text S1 — ASCAT (Allele-Specific Copy number Analysis of Tumors) analysis of tumor ploidy and purity. Text S2. Variant allele frequencies and tumor purity. Text S3. Distinguishing true somatic mutations from false positives using base quality, strand bias and local sequence context. Table S1: The exome cohort used in this study. Table S2: Amount of coding and non-coding variation in each call set, before and after filtering. Table S3: Fraction of sites with unidirectional reads per call set. Table S4: Base quality and sequence context for true and false positives. Table S5: Filtering Single nucleotide variants.(SNVs) using Genome Analysis Tool Kit (GATK) results, percentage mate-pair rescued reads and read depth. Table S6: Partial consensus predictions when the predictions from the third program was below threshold. Figure S1: Number of somatic mutation predictions as a function of tumor ploidy and aberrant cell fraction. Figure S2: Percentage overlap in somatic mutation predictions per sample. Figure S3: Percentage of SNV predictions in each call set for all variants, coding and non-coding. Figure S4: Read depth and allele frequency characteristics for coding and non-coding SNVs in each call set. Figure S5: Read depth and non-reference allele frequency for true and false positives, for all assessed variants. Figure S6: Read depth and non-reference allele frequency for true-positive and false-positive partial-consensus and unique predictions. Figure S7: Fraction of reads mapping to repetitive sequences for true somatic mutations and false positive predictions. Figure S8: Base qualities for true somatic mutations and false positives. Figure S9: Strand bias for true somatic mutations and false positives. Figure S10: GC content for true somatic mutations and false positive predictions. Figure S11: Homopolymer content for true somatic mutations and false positives. Figure S12: Influence of false-positive rates, estimated ploidy, and estimated aberrant cell fraction on number of p [file gm494-S1.docx]

**Supplementary Materials**

**Table of Contents**

1. **Supplementary Text**

S1. Details of ASCAT analysis of tumor ploidy and purity

S2. Variant allele frequencies and tumor purity

S3. Distinguishing true somatic mutations from false positives using base

quality, strand bias and local sequence context

1. **Supplementary Tables**

Table S1: Details of the exome cohort used in this study

Table S2: Amount of coding and non-coding variation in each call set, before and after filtering

Table S3: Fraction of sites with unidirectional reads per call set

Table S4: Comparison of base quality and sequence context metrics between true and false positives

Table S5: Filtering of SNVs using GATK results, percentage mate-pair rescued reads and read depth, for each call set

Table S6: Below threshold predictions from 3rd program for SNV predictions in the partial consensus call sets.

1. **Supplementary Figures**

Figure S1: Number of somatic mutation predictions as a function of tumor

ploidy and aberrant cell fraction

Figure S2: Percentage overlap in somatic mutation predictions per sample

Figure S3: Percentage of SNV predictions in each call set for all variants, coding and non-coding.

Figure S4: Read depth and allele frequency characteristics for all SNVs in each call set compared to coding SNVs in each call set.

Figure S5: Read depth and non-reference allele frequency for true positives and false positives for all assessed variants.

Figure S6: Read depth and non-reference allele frequency for true positives and false positives in the partial consensus and unique predictions.

Figure S7: Distribution of fraction of reads mapping to repetitive sequences for true somatic mutations and false positive predictions.

Figure S8: Distribution of base qualities for true somatic mutations and false positive predictions.

Figure S9: Strand bias for true somatic mutations and false positive predictions.

Figure S10: GC content for true somatic mutations and false positive predictions.

Figure S11: Homopolymer content for true somatic mutations and false positive predictions.

Figure S12: Influence of false positive rates, estimated ploidy and estimated aberrant cell fraction on number of predicted somatic mutations.

**Supplementary Text**

**S1. Details of ASCAT analysis of tumor ploidy and purity**

Estimates of tumor ploidy and aberrant cell fraction were generated using ASCAT (Allele-specific copy number analysis of tumors; Van Loo et al, 2010). Employing the Log-R Ratio and B-allele frequencies across the copy number aberrations present in the genome, ASCAT aims to reach an optimal solution for tumor ploidy, percentage of aberrant cells and a ‘goodness of fit’ estimate for the solution. Plotting predicted ploidy against the number of somatic mutations predicted for each tumor revealed that diploid and near-diploid tumors had similar mutation call rates to predicted tetraploid and near-tetraploid tumors (Figure S1). The two tumors with the greatest number of somatic calls, notably higher than all other tumors, were predicted to be close to triploid with areas of tetraploidy and significant levels of loss of heterozygosity. It may be that these triploid tumors have higher call rates due to higher rates of allelic imbalance, with mutations on the retained alleles having a higher representation in the exome sequencing data. This is compared to tetraploid and near-tetraploid tumors, where both alleles are returned or the retained allele is duplicated and as such many somatic mutations may remain balanced with a wild-type allele.

Expectedly, there is some correlation between the predicted aberrant cell population, however, this is not a strong correlation most likely due to somatic prediction callers being designed in the knowledge that tumor DNA is typically derived from genetically heterogeneous populations and frequently with ‘normal’ (non-aberrant) contamination. Of note, ASCAT cannot accurately estimate the aberrant cell fraction of completely diploid cells, appearing to derive estimates by modeling noise in the data. Therefore for this analysis the percentage of aberrant cells for completely diploid samples was estimated using allele ratios of somatic mutations from both exome data and Sanger sequencing.

Log R Ratios and B-allele frequencies were generated from SNP6 (Affymetrix) CEL files for the tumor-germline pairs using PennCNV (Wang et al, 2007). ASCAT was then used to generate ASCAT profiles in R. The ASCAT profiles for these tumors were generally in agreement with previous analyses; however, ASCAT is noted to have difficulty resolving on a solution when there are subpopulations of cells with different copy number aberrations and when the data is noisy. Additionally, estimation of aberrant cell fraction is problematic in completely diploid cells.

**S2. Variant allele frequencies and tumor purity**

The lack of correlation between non-reference allele frequencies and estimated sample purity (Fig S1C) is inconsistent with expectation that somatic variant frequencies be closely tied to levels of normal contamination with tumor samples. This trend indicates the mutation detection algorithms used here generate large numbers of false positives, particularly when the fraction of reads non-reference allele is low.

Thus, attempts to remove false positive variant calls should improve both the correlation between numbers of variants predicted per sample and non-reference allele frequency (NRAF) and sample purity. We see that this is indeed the case. Filtering out predicted variants with insufficient coverage and/or variant allele frequencies to low for validation by Sanger sequencing (i.e., read depth > 7 in both tumor and germline sample, fraction of read with non-reference allele ≥ 0.2 in the tumor sample and <0.05 in the germline sample) results in an over 3-fold increase in the R-squared value between # of predicted variants per sample and the percentage of aberrant cells predicted by ASCAT (from 0.01163 to 0.03538), as demonstrated in Figure S12A.

Correlation between variant counts and purity is further improved when per sample true positive rates are taken into account. As shown in Figure S1D, both true and false positive rates are related to level of normal contamination in a sample. We calculated the ‘expected’ number of genuine somatic mutations per sample by multiplying true positive rate by total number of predictions from all call sets combined, for each sample. This quantity shows a much stronger correlation with sample purity (r-squared = 0.1404; Figure S12B) than does the total number of raw somatic mutation predictions.

We also observed good correlation (R-squared = 0.196; p-value = 0.025) between median NRAF in the tumor sample and the percentage of aberrant cells predicted by ASCAT after removal of low-quality predictions below the threshold required for Sanger validation (Figure S12C). Partitioning samples by median NRAF is generally consistent with partitioning based on sample purity. All but one of the samples with a purity <60% have a median NRAF below the average of the 27 samples (i.e., < 35.4%), while all but 7 of the samples with purity > 60% have an above average median NRAF. These 7 samples suggest factors other than high false positive rates contribute to the lack of a clear trend between NRAF and purity.

We tested whether correcting for ploidy would clarify the relationship between median NRAF and sample purity in our samples, by stratifying samples by average ploidy as predicted by ASCAT (Figures S12D & S12E). Median NRAF and aberrant cell fraction are correlated for samples with a mean ploidy less than 3 (R-squared = 0.320; p=0.0221; n=16) while samples with a mean ploidy above 3 do not (R-squared = 0.0252; p=0.9414; n=11), indicating the presence of passenger mutations with non-diploid allele frequencies can obscure the relationship between fraction of reads carrying a somatic variant and sample purity.

The assumption that a somatic mutation will be present in 50% of the reads originating from the tumor, and thus to have an allele frequency roughly equal to half of the sample purity, is reasonable for driver mutations affecting oncogenes or tumor suppressors, which are expected to show strong dominant or recessive effects. However, the majority of somatic variants are usually passenger mutations (i.e., play no role in tumor development) and can have allele frequencies that deviate from this expectation due to a number of factors, including technical artifacts, intratumor heterogeneity and local changes in ploidy. No attempt was made to enrich for driver mutations in this study, and therefore most of the mutations identified are likely passengers rather than drivers.

Collectively, these findings suggest the somatic mutation detection algorithms included in this study cannot always adequately account for sample purity or departures from diploid copy-number ratios,affecting variant allele frequencies for reported somatic mutations. This should be taken into consideration when designing strategies to filtering and validating predicted mutations.

**S3. Distinguishing true somatic mutations from false positives using base quality, strand bias and local sequence context**

*Base Quality*

Base quality, which is a scaled estimate of the accuracy of an individual base call at each site within a sequence read, can also be employed to identify genuine sequence variants. For each somatic SNV in our validation set we calculate the median base quality for that site in each read in the tumor-germline pair in which that SNV was identified. Base qualities were significantly lower for false positives (FPs) across reads from both samples, for either allele, with the greatest differences occuring at sites harboring a non-reference allele in the tumor sample (Figure S8, Table S5; Wilcoxon rank-sum p-value < 1e-6).

*Strand Bias*

Additionally, false positives showed greater strand bias than true positives (TPs). Strand bias here is defined as the fraction of reads obtained from the more poorly-covered strand. Ideally, one should obtain similar numbers of reads from each strand, but when there is a bias the fraction of reads coming from the minority strand can be far less than 0.5. Calculating strand bias for each site in our validation set reveals a significant strand bias for the reference allele in FPs, but not so for the non-reference allele (Figure S9, Table S5; Wilcoxon rank-sum p < 1e-5). This effect was restricted to false-positives predictions in the tumor sample only. Predictions that turned out to be germline SNVs did not display a significant difference in strand bias with TPs. As the presence of bidirectional reads is often used to filter putative variants (e.g., Thompson et al 2012, Meyer et al 2013), it is worth noting that a significant proportion of our TPs lacked reads from one of the strands.

*Local sequence context effects*

DNA sequence features are known to influence the power and accuracy of SNV prediction from high-throughput sequence data. For example, reduced local sequence complexity and repetitive sequences can cause mismapping of sequence reads to incorrect locations in the genome, leading to spurious variant calls. The presence of homopolymers is another source of false positives, either indirectly through misalignment of sequence reads or directly through induction of sequencing errors.

To determine whether GC% and the presence of homopolymers were responsible for some of the false positives in our validation data, we examined the complexity of the sequence within a 200 bp window (100bp up- and downstream) of each SNV within our validation set. Sequences surrounding SNVs that failed validation had a significantly higher GC% than somatic variants (Table S5; Wilcoxon rank-sum p < 0.05), while germline variants had higher GC% again (Figure S10). Germline false positive variants (but not those that failed to validate in the tumor) also had significantly higher homopolymer content and likelihood of being found within a homopolmer 3bp or longer than did true somatic variants (Figure S11, Table S5; Fisher Exact p < 0.05).

**Supplementary References**

Meyer JA, Wang J, Hogan LE, Yang JJ, Dandekar S et al. 2013. Relapse-specific mutations in NT5C2 in childhood acute lymphoblastic leukemia. Nature Genetics 45(3):290-4.

Thompson ER, Doyle MA, Ryland GL, Rowley SM, Choong DYH, et al. 2012. Exome Sequencing Identifies Rare Deleterious Mutations in DNA Repair Genes FANCC and BLM as Potential Breast Cancer Susceptibility Alleles. PLoS Genetics 8(9): e1002894.

Van Loo P, Nordgard SH, Lingjærde OC, Russnes HG, Rye IH, et al. 2010. Allele-specific copy number analysis of tumors. Proceedings of the National Academy of Sciences of the United States of America 107: 16910–16915.

Wang K, Li M, Hadley D, Liu R, Glessner J, Grant S, Hakonarson H, Bucan M. 2007. PennCNV: an integrated hidden Markov model designed for high-resolution copy number variation detection in whole-genome SNP genotyping data. Genome Research 17:1665-1674.

**Supplementary Tables**

**Table S1:** ASCAT profiles and sequencing performance summary for the exome cohort

| **Sample** | **Histology** | **ASCAT profile** | | | **Exome Performance Summaries** | | | | | | | |
| --- | --- | --- | --- | --- | --- | --- | --- | --- | --- | --- | --- | --- |
|  |  | Ploidy | Aberrant Cell Fraction (%) | Goodness of Fit (%) | NimbleGen Capture Version | Read Length (bp) | Total Reads (Tumor) | Total Reads (Germline) | % Target bases >=10-fold Coverage (Tumor) | Mean coverage for target bases (Tumor) | % Target bases >=10-fold Coverage (Germline) | Mean coverage for target bases (Germline) |
| 1 | Benign mucinous | 2.01 | 54 | 94.7 | V1 | 75 | 127964720 | 70223228 | 94.43 | 117.58 | 93.66 | 103.79 |
| 2 | Borderline mucinous | 4.23 | 39 | 94.4 | V1 | 75 | 66498560 | 68341690 | 95.62 | 143.64 | 94.8 | 105.74 |
| 3 | Borderline serous | 2.07 | 71 | 98.0 | V2 | 100 | 89329032 | 86985512 | 93.99 | 94.95 | 94 | 113.08 |
| 4 | Borderline serous | 1.98 | 54 | 98.9 | V2 | 100 | 97640008 | 102170126 | 94.85 | 93.29 | 95.87 | 134.2 |
| 5 | Borderline serous | 3.99 | 74 | 99.3 | V2 | 100 | 101472240 | 94963544 | 94.73 | 134.83 | 95.02 | 131.16 |
| 6 | Benign mucinous | 1.98 | 77 | 99.4 | V1 | 75 | 48173894 | 59419426 | 96.06 | 165.5 | 92.94 | 90.37 |
| 7 | Borderline serous | 2.01 | 50 | 98.2 | V2 | 100 | 86316212 | 89529260 | 94.27 | 118.51 | 94.44 | 119.43 |
| 8 | Borderline serous | 4.37 | 26 | 94.2 | V2 | 100 | 86804154 | 80657362 | 94.38 | 117.91 | 94.41 | 114.79 |
| 9 | Benign mucinous | 2.01 | 79 | 93.8 | V2 | 100 | 82655692 | 122614342 | 95.51 | 116.69 | 96.42 | 162.35 |
| 10 | Invasive mucinous | 3.82 | 67 | 92.4 | V2 | 100 | 90924558 | 103094680 | 91.87 | 75.68 | 96.12 | 140.46 |
| 11 | Invasive mucinous | 2.02 | 74 | 97.9 | V2 | 100 | 90271968 | 102117688 | 95.31 | 124.95 | 95.13 | 133.72 |
| 12 | Borderline serous | No suitable model determined | | | V2 | 100 | 98895018 | 107231256 | 94.79 | 94.32 | 95.86 | 131.01 |
| 13 | Borderline serous | 1.99 | 53 | 98.8 | V2 | 100 | 104554942 | 112453072 | 91.08 | 47.86 | 95.27 | 108.95 |
| 14 | Borderline serous | 2.19 | 39 | 95.1 | V2 | 100 | 129491814 | 142609794 | 96.59 | 186.42 | 96.64 | 187.86 |
| 15 | Borderline serous | 2.81 | 31 | 97.1 | V2 | 100 | 86200738 | 94405108 | 94.23 | 116.01 | 93.65 | 104.97 |
| 16 | Invasive mucinous | 2.13 | 83 | 98.4 | V2 | 100 | 92729982 | 86972104 | 94.36 | 89.44 | 95.33 | 119.61 |
| 17 | Borderline mucinous | 1.98 | 68 | 99.3 | V2 | 100 | 125754244 | 122838464 | 96.2 | 158.19 | 95.64 | 147.25 |
| 18 | Invasive mucinous | 2.32 | 84 | 99.0 | V2 | 100 | 92942400 | 108949244 | 96.45 | 177.32 | 95.57 | 148.47 |
| 19 | Borderline serous | 4.14 | 68 | 97.1 | V2 | 100 | 88327834 | 102311750 | 94.14 | 115.51 | 94.36 | 113.17 |
| 20 | Invasive mucinous | 2.01 | 81 | 99.2 | V2 | 100 | 107496432 | 144887088 | 89.11 | 54.58 | 96.33 | 188.6 |
| 21 | Invasive mucinous | 2.29 | 87 | 99.0 | V2 | 100 | 190223336 | 119515304 | 94.84 | 102.31 | 95.72 | 150.32 |
| 22 | Invasive mucinous | 3.90 | 59 | 98.8 | V2 | 100 | 124862496 | 116707640 | 94.94 | 125.2 | 96.37 | 154.75 |
| 23 | Invasive mucinous | 3.99 | 66 | 97.9 | V2 | 100 | 115757886 | 117160454 | 92.73 | 93.04 | 96.24 | 152.75 |
| 24 | Invasive mucinous | 2.00 | 74 | 99.5 | V2 | 100 | 89192606 | 123853880 | 95.15 | 120.38 | 96.65 | 165.1 |
| 25 | Borderline mucinous | 4.22 | 80 | 98.9 | V1 | 75 | 62641994 | 61934484 | 95.25 | 137.8 | 88.75 | 83.63 |
| 26 | Invasive mucinous | 3.11 | 67 | 98.0 | V2 | 100 | 108378824 | 101338470 | 94.94 | 108.77 | 95.69 | 135.26 |
| 27 | Invasive mucinous | 3.12 | 72 | 98.2 | V2 | 100 | 98017724 | 109985782 | 94.09 | 106.9 | 96.13 | 142.44 |

**Table S2**: Predicted coding and non-coding variants in each call set, before and after filtering

|  | **MJS** | **MJ** | **MS** | **JS** | **M** | **J** | **S** | **TOTAL** |
| --- | --- | --- | --- | --- | --- | --- | --- | --- |
| **All predicted SNVs** | 1483 (16%) | 83 (1%) | 462 (5%) | 298 (3%) | 1756 (19%) | 2387 (26%) | 2757 (30%) | 9226 |
| **All coding SNVs** | 908 (61%) | 39 (47%) | 124 (27%) | 148 (50%) | 548 (31%) | 1535 (64%) | 1210 (44%) | 4512 |
| **All non-coding SNVs** | 575 (39%) | 44 (53%) | 338 (73%) | 150 (50%) | 1208 (69%) | 852 (36%) | 1547 (56%) | 4714 |
| **Filtered SNVs** | 1385 (54%) | 16 (1%) | 370 (15%) | 57 (2%) | 279 (11%) | 80 (3%) | 360 (14%) | 2547 |
| **Filtered coding SNVs** | 839 (61%) | 6 (38%) | 99 (27%) | 23 (40%) | 36 (13%) | 34 (43%) | 85 (24%) | 1122 |
| **Filtered non-coding SNVs** | 546 (39%) | 10 (63%) | 271 (73%) | 34 (60%) | 243 (87%) | 46 (58%) | 275 (76%) | 1425 |
| **Average filtered SNVs/sample (Range)** | 20.2 (1-246) | 0.4 (0-6) | 10.0 (5-42) | 1.3 (0-11) | 9.0 (3-33) | 1.7 (0-23) | 10.2 (5-25) |  |
| **# SNVs filtered out (% all predicted SNVs)** | 98 (7%) | 67 (81%) | 92 (20%) | 241 (81%) | 1477 (84%) | 2307 (97%) | 2397 (87%) | 6679 (72%) |

**Table S3**: Fraction of sites covered by unidirectional reads only, per call set.

|  | **MJS** | **MJ** | **MS** | **JS** | **M** | **J** | **S** |
| --- | --- | --- | --- | --- | --- | --- | --- |
| **All predicted SNVs** | 1483 | 83 | 462 | 298 | 1756 | 2387 | 2757 |
| **All SNVs only covered by**  **uni directional reads** | 113 (8%) | 33 (40%) | 174 (38%) | 48 (16%) | 1107 (63%) | 1008 (42%) | 492 (18%) |
| **Filtered SNVs** | 1385 | 16 | 370 | 57 | 279 | 80 | 360 |
| **Filtered SNVs only covered by uni directional reads** | 98 (7%) | 10 (63%) | 134 (36%) | 18 (32%) | 209 (75%) | 61 (76%) | 124 (34%) |

**Table S4**: Comparison of sequence context, base quality and strand bias between true and false positives.

| **Feature** | **All True Positives** | **All False Positives** | **Did Not Validate** | **Germline** |
| --- | --- | --- | --- | --- |
| **Median %GC (SNV+/-100bp)^1^** | 51% | 56%* | 53%* | 60%* |
| **Median # adjacent sites in homopolymers (SNV+/-100bp)^2^** | 93 | 96* | 95 | 101* |
| **Fraction of SNVs found in homopolymer (2+)^2^** | 0.367 | 0.368 | 0.35 | 0.41 |
| **Fraction of SNVs found in homopolymer (3+)^2^** | 0.057 | 0.103 | 0.113 | 0.077* |
|  |  |  |  |  |
| **Median Base quality^3^** |  |  |  |  |
| - germline reference allele | 37 | 36 | 36* | 36* |
| - germline alternate allele | 0 | 0 | 0* | 0* |
| - tumor reference allele | 37 | 36 | 36* | 37 |
| - tumor alternate allele | 25 | 22* | 19.5** | 24 |
| **Strand ratio (Strand bias)^4^** |  |  |  |  |
| - germline reference allele | 0.39 | 0.3** | 0.26** | 0.38 |
| - germline alternate allele | 0.0 | 0.0 | 0.0 | 0.0 |
| - tumor reference allele | 0.39 | 0.28** | 0.26** | 0.33 |
| - tumor alternate allele | 0.37 | 0.33 | 0.33 | 0.29 |

Number of asterices indicates level of statistical significance: ****p < 1e-15, ***p < 1e-10, **p < 1e-5, *p < 0.05. Significance was tested using Wilcoxon rank-sum test for continuous variables and Fisher’s Exact Test for fraction of SNVs with adjacent to homopolymers.

^1^Percent GC was measured in the 200 bp surrounding SNVs in the validation set (100 bp up- and downstream of the SNV). ^2^Homopolymers were defined as the same nucleotide appearing two/three or more times. Number of adjacent sites in homopolymers was taken from the 200 bp surrounding each SNV in the validation set as well. “Fraction SNVs adjacent to homopolymer” measured the fraction of SNVs found at the end of a homopolymer run.

^3^Median base quality score for non-reference base calls in the tumor samples was obtained using SAMtools.

^4^Strand bias is the fraction of reads from the strand with lower coverage, be that the + or – strand. In the absence of any strand bias, this value should be very close to 0.5.**Table S5**: Additional filtering of SNVs outside of the full consensus call set.

| **Somatic Mutation Prediction Feature** | **2 caller consensus validation rate** | | | **No consensus validation rate** | | | **Overall validation rate** | **True positive dropout rate** |
| --- | --- | --- | --- | --- | --- | --- | --- | --- |
|  | MJ | MS | JS | M | J | S |  |  |
| Base validation rate | 5/13 (38.5%) | 29/37 (78.4%) | 10/28 (35.7%) | 4/31 (12.9%) | 1/26 (3.8%) | 1/47 (2.1%) | 50/183 (27%) |  |
| GATK prediction for SNV in tumor | 5/7 (71.4%) | 29/36 (80.5%) | 9/23 (39.1%) | 3/16 (18.7%) | 1/9 (11.1%) | 1/20 (5%) | 48/113 (42%) | 4% |
| % mate-rescued reads <7% | 5/7 (71.4%) | 29/37 (78.4%) | 10/20 (50%) | 4/25 (16%) | 1/6 (14.3%) | 1/38 (2.6%) | 50/133 (38%) | 0% |
| GATK + mate-rescued | 5/5 (100%) | 29/36 (80.5%) | 9/15 (60%) | 3/14 (21.4%) | 1/2 (50%) | 1/15 (6.7%) | 48/87 (55%) | 4% |
| RD >10 (T & G) | 5/13 (38.5%) | 25/28 (89.2%) | 10/27 (37%) | 3/14 (21.4%) | 1/26 (3.8%) | 1/32 (3%) | 45/140 (32%) | 10% |
| RD >15 (T & G) | 5/13 (38.5%) | 18/18 (100%) | 10/26 (38.1%) | 1/9 (11.1%) | 1/26 (3.8%) | 1/19 (5.3%) | 36/111 (32%) | 28% |
| GATK + mate-rescued + RD >10 | 5/5 (100%) | 25/27 (90.1%) | 9/14 (64.3%) | 2/6 (33.3%) | 1/2 (50%) | 1/11 (9.1%) | 43/65 (66%) | 14% |
| 3rd somatic caller with lowered thresholds | 4/6 (67%) | 20/20 (100%) | 9/26 (34.6%) |  |  |  | 33/52 (63%) | 34% |
| GATK + 3rd somatic caller with lowered thresholds | 4/6 (67%) | 20/20 (100%) | 9/23 (39.1%) |  |  |  | 33/49 (67%) | 34% |

Validation rate = true positives/total SNVs assessed.

^1^Partial consensus predictions (made by two programs).

^2^No consensus predictions (made by only one program).

^3^‘True positive dropout’ is percentage of true positives that would be discarded if the indicated set of filters was applied, i.e., loss in sensitivity.

^4^‘Base validation rate’ refers to positive predictive values prior to filtering.

^5^Filtering on percentage of reads mapped from mate-rescue.

^6^Filtering on read depth (RD) increased to 10 or 15 reads in tumor and germline.

^7^Non-reference allele frequencies – tumor frequency increased from ≥0.2, germline decreased from ≤0.03.

^8^Variant covered by reads from both directions – bidirectional evidence.

^9^Filtering based on variants being predicted by one of the other programs at values lower than those used for the original call set thresholds.

^10^Filtering on SNV predicted in the tumor but not the germline by GATK’s Unified Genotyper

**Table S6**: Below threshold predictions from 3rd program for SNV predictions in the partial consensus call sets.

| **Partial Consensus Call Set** | **Total # of predictions in validation set** | **Overall validation rate** | **3rd Program** | **# with predictions below threshold in 3rd program** | **Validation rate of predictions where 3rd program was below threshold** | **True positive dropout rate** |
| --- | --- | --- | --- | --- | --- | --- |
|  |  |  |  |  |  |  |
| JointSNVMix2 & SomaticSniper | 28 | 10/28 (35.7%) | MuTect | 26 | 9/26 (34.6%) | 1/10 (10%) |
|  |  |  |  |  |  |  |
| MuTect & JointSNVMix2 | 13 | 5/13 (38.5%) | SomaticSniper | 6 | 4/6 (66.6%) | 1/5 (20%) |
|  |  |  |  |  |  |  |
| MuTect & SomaticSniper | 37 | 29/37 (78.4%) | JointSNVMix2 | 36 | 28/36 (77.8%) | 1/29 (3.4%) |

Validation rate = # true positives/total # of SNVs we attempted to validate. Both the validation rate in our validation set and the validation rate that would be obtained if a prediction from the 3^rd^ program, at any threshold, had been required for taking a SNV to the validation step. True positive dropout rate gives the number and percentage of true positives that would have been missed if a prediction from the 3^rd^ program had been required.

For each program ‘below threshold’ is defined as follows: for MuTect, any prediction with a ‘REJECT’ flag; for SomaticSniper, any prediction with a Somatic Score < 40 but >= 15, and for JointSNVMix2, any prediction with a non-zero probability of p_AA_AB | p_AA_BB.

**Supplementary Figures**

**(A)**

**(B)**

**(C)**


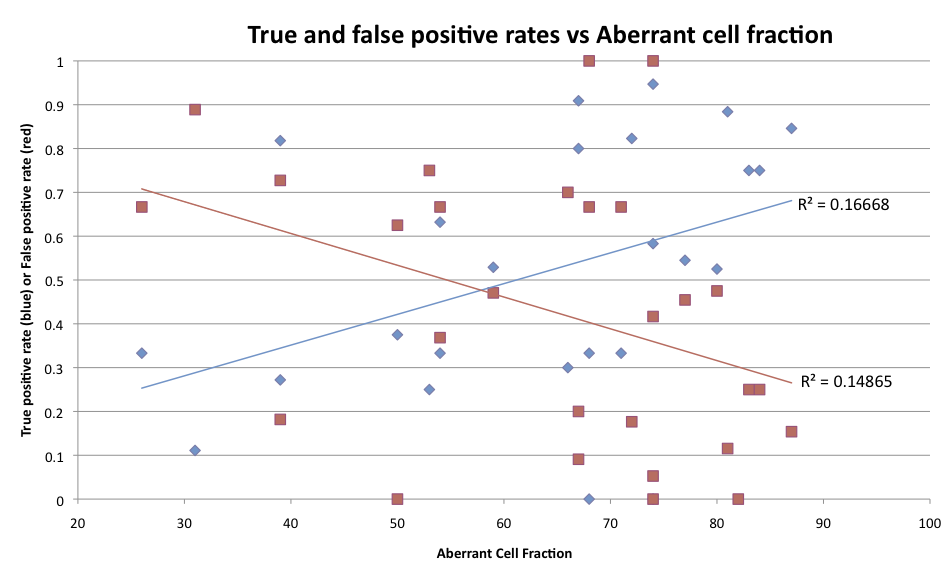


**Figure S1**: Number of somatic SNV predictions as a function of tumor ploidy (A) and aberrant cell fraction (B), as calculated using ASCAT. (C) The true positive and false positive rates as functions of aberrant cell fraction. Increased sample purity improves both true and false positive rates.

**(A)**


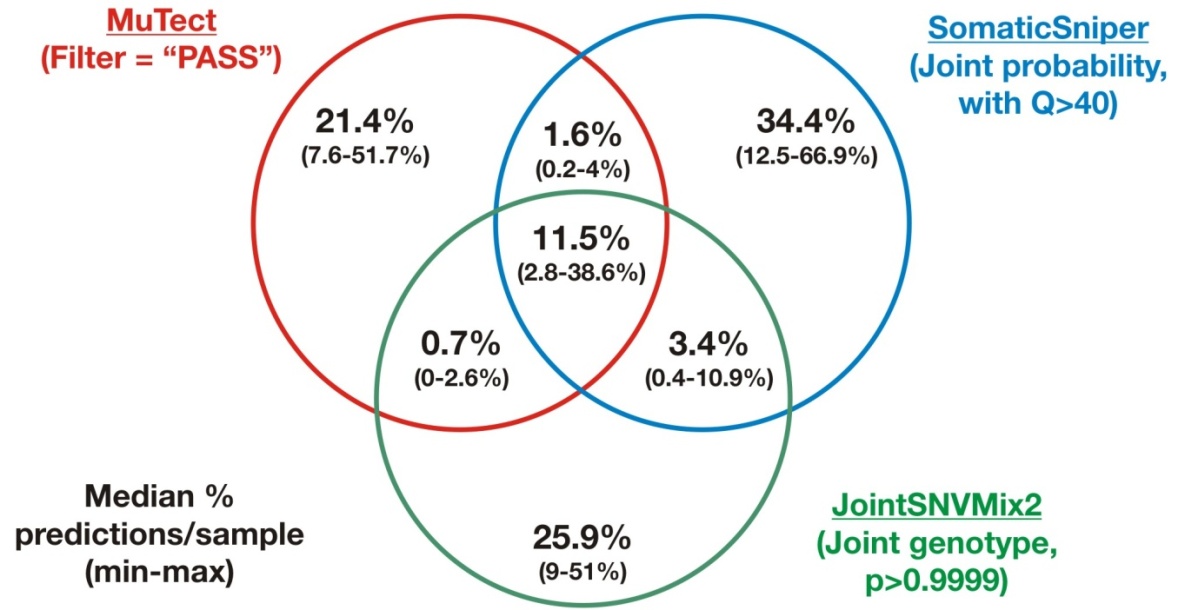


**(B)**

**Figure S2**: Median, minimum and maximum per sample percentage overlap in somatic SNV predictions for (A) all predictions from each program for each sample and (B) for predictions after filtering out mutations that would unlikely to validate using Sanger technology [Methods].


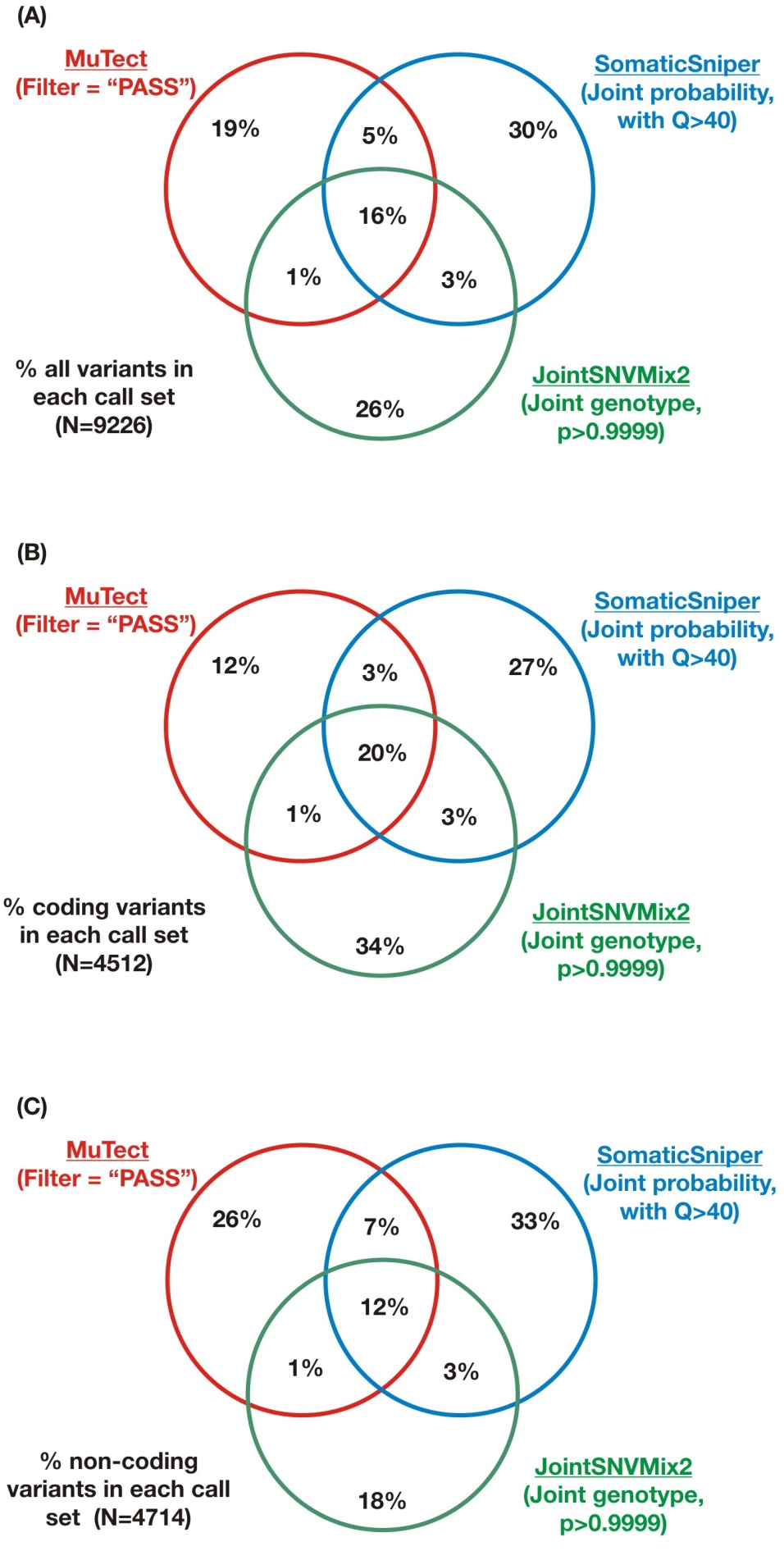


**Figure S3:** Percentage of predicted variants in each call set. (A) All variants, (B) Coding variants, (C) Non-coding variants. Although the three program (MJS) consensus is slightly lower in the non-coding variants, overall the trends for the coding and non-coding groups hold to those observed for all variants combined.

**(A)** **(B)**

**(C)** **(D)**

**(E)** **(F)**

**(G)** **(H)**

**Figure S4:** Call set characteristics. Removal of the non-coding variants was found not to significantly alter the read depth (A-D) and non-reference allele frequency (E-H) characteristics observed for each call set for all variants combined.

**(A)**

**(B)**

**Figure S5**: Read depth and non-reference allele frequency for true positives and false positives for all assessed variants. Read depth coverage (A) and non-reference allele frequency (B) for true positive (TP) and false positive predictions (Did Not Validate (DNV) or Germline (G)) in tumor (_T) and germline (_G) samples. The median for each group is given below.

**(A)**

**(B)**

**Figure S6**: Read depth and non-reference allele frequency for true positives and false positives for the partial consensus and unique predictions. Read depth coverage (A) and non-reference allele frequency (B) for true positive (TP) and false positive predictions (Did Not Validate (DNV) or Germline (G)) in tumor (_T) and germline (_G) samples. The median for each group is given below the x-axis.

**(**


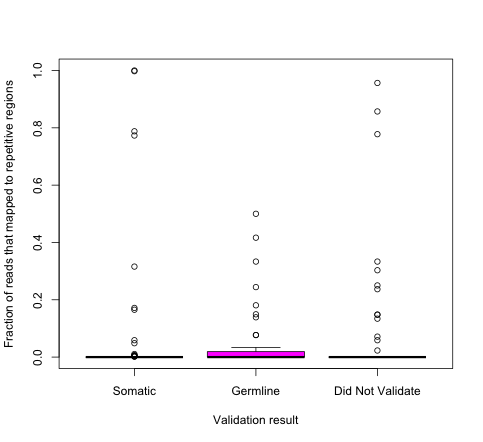


**Figure S7**: Distribution of fraction reads mapping to repetitive sequences for true somatic variants and false positive predictions. False positive SNVs have been divided into those that were found in the germline sample during validation (Germline) and those that were not detected in the tumor or the germline during validation (Did Not Validate). The whiskers on the plot represent values within 1.5 times the interquartile range (IQR) plus/minus the boundaries of the IQR, while open circles represent outliers – values that exceed those thresholds.**
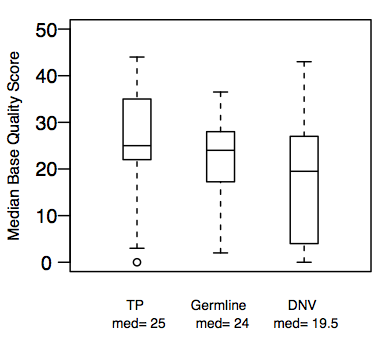
**

**Figure S8**: Distribution of base qualities as reported by SAMtools, of non-reference base calls in the tumor samples, for true somatic mutations and false positive predictions.


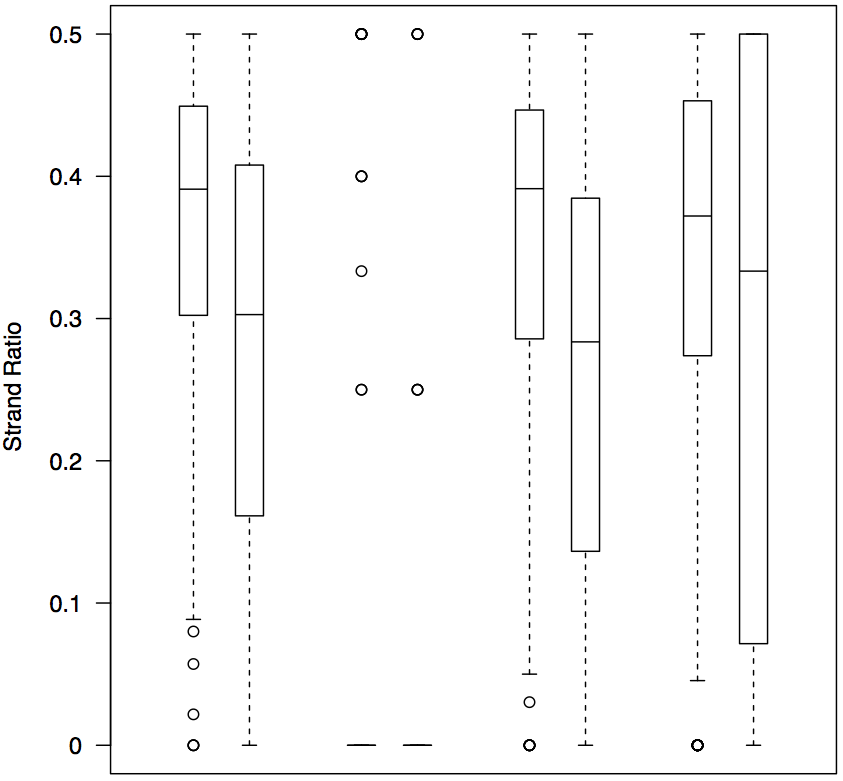


TP FP TP FP TP FP TP FP

Normal Normal Tumor Tumor

Reference Non-Reference Reference Non-Reference

**Figure S9**: Strand bias for true somatic mutations and false positive predictions. Strand bias for TPs and FPs in the germline (left half of plot) and tumor (right half of plot). The first boxplot within each half is for the reference allele and the second boxplot within each half is for the non-reference allele. Strand bias is the fraction of reads that come from the strand with lower coverage, be that the + or – strand. In the absence of any strand bias, this value should be very close to 0.5.


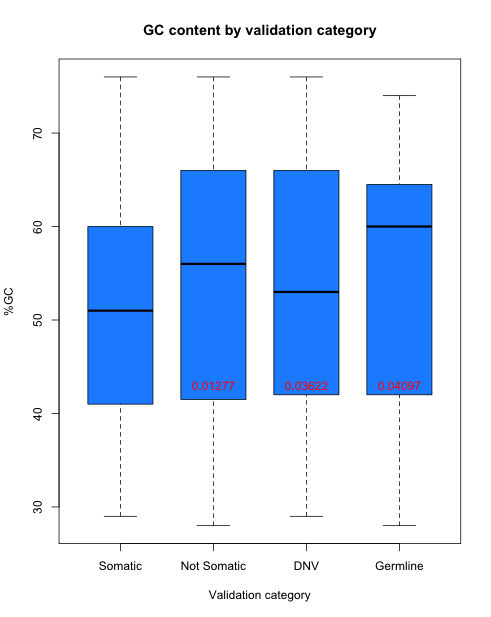


**Figure S10**: GC content for true somatic variants and false positive predictions. Distribution of percent GC in the 200 bp surrounding SNVs in the validation set, by validation result. Values in red are p-values from a one-tailed Wilcoxon rank-sum test comparing SNVs in that class to validated somatic mutations.


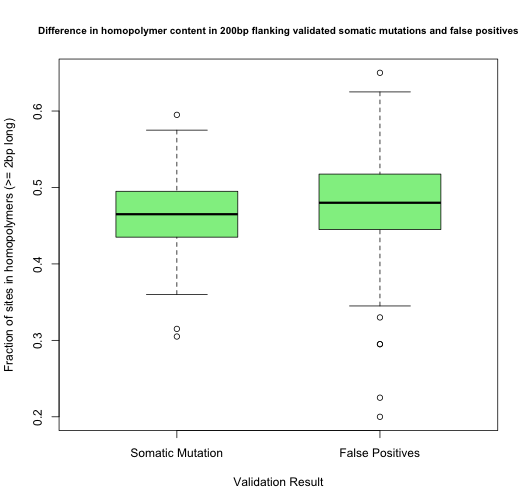


**Figure S11**: Fraction of the 200 bp surrounding each SNV in the validation set occurring in homopolymers, i.e., the same nucleotide appearing two or more times, for true somatic variants and false positive predictions. The whiskers on the plot represent 1.5 times the interquartile range (IQR) plus/minus the boundaries of the IQR, while open circles represent outliers – values that exceed those thresholds.

**(A)**

**(B)**

**(C )**

**(D)**

**(E)**

**Figure S12**: Influence of false positive rates, estimated ploidy and estimated aberrant cell fraction on predicted somatic frequencies. (A) Number of predicted somatic point mutations not suitable for Sanger sequencing (read depth > 7 in both tumor and germline sample, fraction of read with non-reference allele ≥ 0.2 in the tumor sample and <0.05 in the germline sample), across all call sets, and tumor purity, per sample. (B) Expected number of true somatic point mutations and tumor purity per sample. Expected number of true mutations was calculated as sample true positive rate [fraction of mutations tested that validated] multiplied by the total number of somatic mutation predictions per sample, across all call sets. (C) Median non-reference allele frequency (NRAF) of all predicted mutations suitable for Sanger validation and tumor purity, per sample. (D) Median NRAF and tumor purity per sample, for samples with mean ploidy < 3 or (E) mean ploidy ≤3 (bottom), where mean ploidy is estimated by ASCAT.
